# On Treatment of Ascites by Intra Peritoneal Injections

**Published:** 1853-06

**Authors:** 


### ECLECTIC DEPARTMENT,

### AND SPIRIT OF THE MEDICAL PERIODICAL PRESS.

---

*On treatment of Ascites by Intra-peritoneal Injections.* By M. Velpeau.

A question of great importance from its novelty and the limited number of statistical data, which we as yet possess, to test its validity, and the cases to which it may be curatively applicable, is the late treatment of ascites by intra-peritoneal injections of iodine. The use of ioduretted injections, first introduced into practice by M. Velpeau in the treatment of hydrocele, has proved their utility in the removal of serous effusions, and their superiority over various injections; and seems on the eve of becoming generalized as a treatment applicable to all serous effusions of a chronic character. One would *a priori* think, that the startling proposition to inject a fluid so irritant as the tincture of iodine into a cavity lined by so extensive and delicate a membrane as the abdominal, would never be tried; we would at once anticipate, as the direct and inevitable consequence, a general and fatal peritonitis. Yet, since facts are beginning to multiply on this subject, although they do not prove the utter harmlessness of the procedure, they demonstrate the radical cure of cases, wherein all other treatment had failed; objections, however plausable and urgent they may theoretically appear, must give way to experiments and facts. As a matter of course, this treatment cannot be applicable to all cases; as yet, statistical observation has not been sufficiently extended to point out accurately those cases to which this mode of treatment might be specially applicable. Ascites being but a symptom, various are the organic lesions of which it is the effect; diseases of the heart, especially of its right chambers, offering impediments to the ingress of venous blood; diseases of the liver, particularly those causing atrophy of its tissue, interfering with venous return, as cirrhosis; diseases of the spleen, especially those of a miasmatic nature, the sequelæ of protracted intermittents, and renal diseases; where the dropsy is a consequence of cardiac and renal disease, it is of a general nature. I do not believe that ascites, the consequence of these organic lesions, can receive any benefit from ioduretted treatment, inasmuch as the cause is usually irremediable and permanent, and therefore so must be the effect. Yet, there are unquestionably cases of ascites, which might be designated simple, as that resulting from general anemia; that the effect of a peritonitis acute or sub-acute, where the inflammatory peritoneal action having been subdued, the serous effusion yet remains, and that sometimes after all treatment for its relief had proved nugatory. In the former of these cases, where a well tried tonic treatment with chalybeates shall have failed, associated with drastic and saline cathartics and diuretics, the injections of iodine may succeed, as also in ascites, the result of an acute or subacute peritonitis; here inflammatory action may have been completely subdued, and we have to do with its consequences, its effused products. In these cases, as well as in ascites, the result of prolonged and invincible anemia, it would seem in the generality of cases, that the pathological action which maintains the dropsy, is merely the predominance of exhalation over absorption, there being probably no organic alteration persisting otherwise to account for its continuance; here I can see from the analogy of its action in hydrocele, that from ioduretted injections a new action being originated in the peritoneal surface, an impetus is given to absorption, gradually the effused fluid is observed, exhalation by the same rests in abeyance, and thus an equilibrium being established between exhalation and absorption, a radical cure is effected. The opinion that the irritant influence of iodine necessarily produces, when injected into the abdominal cavity, an extensive and fatal peritonitis, appears to be more apparent than real. In corroboration of the truth of this statement, I herewith furnish you some interesting facts, taken from published cases, treated in Hotel Dieu, of Lyons, by M. Teissier, one of the attending physicians.

"I have practiced, says he, up to the present time, the intra-peritoneal injection of iodine upon six female patients affected with ascites. Of this number, two have been cured; with the third, the result is yet uncertain, because the injection is recent; but there have been no accidents, and the consequences of the injection are to the present moment favorable; with two others, the operation has been *innocent* though *unsuccessful,* the ascites having been reproduced a short time after; with the sixth patient, symptoms of peritonitis rapidly followed, and the patient died in forty-eight hours. In this fatal case I had to do with a young woman twenty-two years old, in the last stage of scrofulous cachexia, and whose case offered no chance of success by any other method. Thus: two patients cured; their ascites having resisted all ordinary means, purgatives, diuretics, vesicatories, mercurial ointment; one patient, result doubtful; one operation perfectly *innocent* but *unsuccessful;* one operation followed by death. I ought to say, in this last operation, it was impossible to make the injection run out, after having been thrown in. The injection in these cases was composed of iodine, iodide of potassium, and water. It appears, then, evident to me, that this operation is destined to render great service, but it will not always be innocent. MM. Gromier and Teissier."

The paragraph which concludes these statistical results of MM. Gromier and Teissier, in my estimation, imports the true appreciation which should be accorded to the use of these injections in cases of confirmed and invincible ascites. In the last case of M. Teissier, the ascites may have been the consequence of a tubercular peritonitis, as he remarks that the patient was in the last stage of scrofulous cachexia, though he makes no mention of this lesion as having been the cause of the dropsy. These injections of iodine have been successfully used in certain cases of ovarian dropsy, as the unilocular form, and have been advised, as you are aware, in chronic and invincible diseases of joints, particularly in chronic synovitis. I shall not here dwell longer on this subject, as I shall resume its consideration in my next letter, and may then be able to furnish you with some statistical details.—*Dr. E. F. Smith, European Corres. of St. Louis Med. and Surg. Jour.*

*Is not Blood-letting sometimes dangerous in Apoplexy?* By M. AUSSAGUEL.

This question assuredly merits the attention of every practitioner. M. Aussaguel has collected in his inaugural thesis, from which we borrow this extract, a number of facts upon the subject, which demand grave consideration.

"M. Cruveilhier, when he lectures upon the treatment of cerebral hæmorrhage, never fails to say, 'undoubtedly it is necessary to bleed, but be very circumspect,' * * and then he relates candidly, that having been sent for to visit a patient in the city, whom he found threatened with an apoplectic attack, he hastened to open a vein; the wound was scarcely closed when the patient was attacked with hemiplegia; and he adds, 'the relatives of the patient did not hesitate to say that it was my lancet that had done the mischief.'

"Since then we have read the thesis of M. Cornil: 'A woman,' says he, 'whom I observed last year in M. Rostan's ward, was occupied with her household duties, when she experienced, all at once, a loss of power in her left upper and lower extremity. She with difficulty walked to the house of her physician, who bled her immediately. After the venesection, she was unable to rise: she was completely hemiplegic.'

"The following instance came under our own observation. A friend came to us, stammering in such a manner, that he required fifteen minutes to make us understand that, the morning of the same day, upon awakening, he was greatly surprised to find himself in this condition. There was slight loss of power in the right arm, and its sensibility was diminished. Dr. Batailhé having been summoned, practiced venesection. The next day the stammering had increased, and the patient was copiously bled a second time. Syncope ensued, and the patient revived in fifteen minutes in a state of complete hemiplegia. It is now two years since he was able to utter a word.

"I ask, then, if facts of this kind were numerous, would they not have a kind of accusing eloquence against the employment of blood-letting? and when an impartial witness observes their development, is he not tempted to say, with the relations of M. Cruveilhier's patient, 'it is the lancet that has done the mischief?'"

The author afterward attempts to account for these exceptional cases, and his explanations are not without a certain value.

"What occurs after blood-letting in certain cases of pneumonia? Does not the weak, small pulse, become full, strong, and well developed? Do we not observe an increase in the forces of economy, and is it not generally believed that at that moment another congestive movement toward the lung occurs? Therefore it is to combat the results of blood-letting by blood-letting itself, that M. Bouillaud advises repeated venesection; in other words, the loss of the same quantity of blood is of greater efficacy when it is abstracted by several operations than by one.

"Should we wonder if this were true also for the brain? Why wonder that this organ, inclosed in its unyielding case, engorged with blood, resists the tendency to hæmorrhage for a time, and then yields to it after venesection, the circulation at that moment becoming more active? In other words, are there not two distinct causes operating in the production of apoplexy; the circulating mass and the power which propels it? and does it not seem impossible to diminish the one without increasing the other?

"To diminish the first, without increasing the second, such should be the aim of the practitioner.

"It is with a view to attain this end that we propose that a vein should never be opened until the head of the patient is elevated, and cold applications are made to it, and the blood is invited to the lower extremities by sinapisms or pediluvia, and the patient has taken some soothing draught, with a few drops of digitalis."—*Revue Med. Chir. de Malgaigne; from Virginia Med. and Surg. Jour.*

